# Anti-HIV-1 Activity of Flavonoid Myricetin on HIV-1 Infection in a Dual-Chamber *In Vitro* Model

**DOI:** 10.1371/journal.pone.0115323

**Published:** 2014-12-29

**Authors:** Silvana Pasetto, Vanessa Pardi, Ramiro Mendonça Murata

**Affiliations:** University of Southern California, Ostrow School of Dentistry, Division of Periodontology Diagnostic Sciences, Dental Hygiene & Biomedical Science, Los Angeles, California, United States of America; University of Colorado Denver, United States of America

## Abstract

HIV infection by sexual transmission remains an enormous global health concern. More than 1 million new infections among women occur annually. Microbicides represent a promising prevention strategy that women can easily control. Among emerging therapies, natural small molecules such as flavonoids are an important source of new active substances. In this study we report the *in vitro* cytotoxicity and anti-HIV-1 and microbicide activity of the following flavonoids: Myricetin, Quercetin and Pinocembrin. Cytotoxicity tests were conducted on TZM-bl, HeLa, PBMC, and H9 cell cultures using 0.01–100 µM concentrations. Myricetin presented the lowest toxic effect, with Quercetin and Pinocembrin relatively more toxic. The anti-HIV-1 activity was tested with TZM-bl cell plus HIV-1 BaL (R5 tropic), H9 and PBMC cells plus HIV-1 MN (X4 tropic), and the dual tropic (X4R5) HIV-1 89.6. All flavonoids showed anti-HIV activity, although Myricetin was more effective than Quercetin or Pinocembrin. In TZM-bl cells, Myricetin inhibited ≥90% of HIV-1 BaL infection. The results were confirmed by quantification of HIV-1 p24 antigen in supernatant from H9 and PBMC cells following flavonoid treatment. In H9 and PBMC cells infected by HIV-1 MN and HIV-1 89.6, Myricetin showed more than 80% anti-HIV activity. Quercetin and Pinocembrin presented modest anti-HIV activity in all experiments. Myricetin activity was tested against HIV-RT and inhibited the enzyme by 49%. Microbicide activities were evaluated using a dual-chamber female genital tract model. In the *in vitro* microbicide activity model, Myricetin showed promising results against different strains of HIV-1 while also showing insignificant cytotoxic effects. Further studies of Myricetin should be performed to identify its molecular targets in order to provide a solid biological foundation for translational research.

## Introduction

The human immunodeficiency virus (HIV) is responsible for the acquired immunodeficiency syndrome (AIDS) disease [1], [2]. The first cases of AIDS were reported in 1981 and today, more than 30 years later, it is one of the world's most serious health problems. There are approximately 35 million people currently living with HIV [1], [2]. Even with all the information that has become available, sexual transmission of HIV-1 remains an important route of infection [3], [4]. Although female to male transmission of HIV can occur, the majority of cases (80%) involve transmission of virus from male to female [5]. Women are more vulnerable to HIV infection by sexual transmission due to biological, economic, and cultural factors [6]–[8]. The development of potent and safe topical anti-HIV formulations, referred to as microbicides, has become a priority in HIV research [9]. Currently available HIV prevention techniques are often not feasible for many women living in poor resource settings. The availability of microbicides would greatly empower women to protect themselves and their partners. Unlike male or female condoms, microbicides are a potential preventive option that women can easily control since they do not require the cooperation, consent, or even knowledge of the partner [10]. Among emerging therapies, natural small molecules have not received sufficient attention. Phytochemical investigations have resulted in isolation and identification of potentially bioactive flavonoids such as Myricetin, Quercetin and Pinocembrin. These flavonoids are present in most plants tissues [11], [12] and present anti-viral [13], antioxidant [14]–[16], antibacterial and anti-inflammatory [17], as well as other pharmacological activities, while also having low toxicity in eukaryotic cells [18]. However, the effect of these natural compounds as a potential microbicides against HIV has yet to be determined.

The aims of this *in vitro* study were I) to evaluate the cytotoxicity/anti-HIV-1 activity of Myricetin, Quercetin, and Pinocembrin and II) to determine the anti-HIV-1 activity of these flavonoids using a dual-chamber model.

## Materials and Methods

### Flavonoids

**Figure 1:**
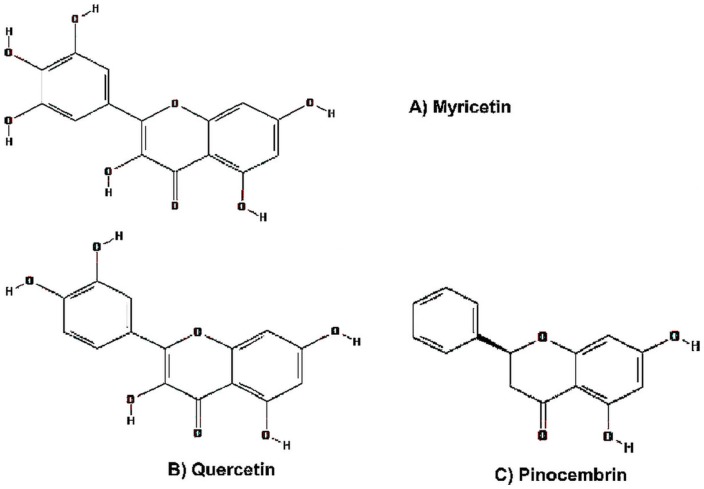
Structural formulae of: A) Myricetin, B) Quercetin, C) Pinocembrin. Compound ID: 5281672; ID: 5280343; ID: 68071.

The structures of the flavonoids used in this study, Myricetin, Quercetin, and Pinocembrin, are shown in Fig. 1. All were obtained from Sigma Aldrich (St Louis, USA). They were prepared in Dimethyl sulfoxide (DMSO) (Sigma-Aldrich, St Louis, MO) at concentration of 0.01–100 µM and added to the cultured cells (TZM-bl, HeLa, H9, and PBMC). The positive control treatment was Zidovudine (AZT) (Sigma Aldrich, St Louis, USA). The AZT concentration was established at 60 µM after cytotoxicity assay on TZM-bl, HeLa, PBMC, and H9 cells (0.06 µM–6000 µM) and literature consultation. Negative controls included untreated cells and cells treated with vehicle alone (1% DMSO, v/v; Sigma Aldrich, St Louis, USA).

### Cell lines

TZM-bl (previously named JC53-b) is a HeLa cell line genetically engineered to express CD4, CCR5, and CXCR4. It contains integrated reporter genes for firefly luciferase and *E. coli* β-galactosidase under the control of a HIV-1 long terminal repeat (LTR). This allows quantification of HIV infectivity because TZM-bl cells are very sensitive to HIV-1 infection. The luciferase activity is easily quantified by luminescence, and is directly proportional to the number of infectious virus particles present in the inoculum [19], [20]. In this study, TZM-bl cells were used as a target for HIV-1 BaL infection. The H9 cell is a single-cell clone derived from a specific HUT 78 cell line, a human cutaneous T cell lymphoma derived from the peripheral blood that exhibits high-yield permissive growth of HIV-1. H9 cells express CD4 receptor and CXCR4 co-receptor. The TZM-bl and H9 cells were obtained through the NIH AIDS Reagent Program, Division of AIDS, NIAID, NIH. The commercially available PBMC cells (Astarte Biologics, LLC, Redmond, USA, Catalogue number 1001, Lot number 2536DE13) were collected from healthy donors by leukapheresis, and further purified on Ficoll-sodium metrizoate density gradients. The PBMC sample used for our analysis was from a healthy 47-year-old Caucasian male (vendor information). The HeLa cell line (ATCC CCL-2, Manassas, VA. USA) was derived from a female cervical tract and used in the dual-chamber epithelial model. The TZM and HeLa cells were cultured with DEMEM and 10% heat-inactivated Fetal Bovine Serum (FBS) (Lonza Walkersville, Inc. Walkersville, MD) at 37°C in humid air containing 5% CO_2_. The H9 cells were grown in RPMI-1640 medium, supplemented with 10% FBS at 37°C in humid air containing 5% CO_2_. Isolated human PBMC cells were grown for 3 days in RPMI-1640, supplemented with FBS 15% (v/v) and 2 µg/mL of phytohemagglutinin - PHA (Sigma-Aldrich, St Louis, MO), then stimulated with 50 U/ml human IL-2 (Roche Diagnostics, Indianapolis, IN) as described by Li et al. [21].

### Virus Strains

Three virus strains were obtained through the NIH AIDS Reagent Program, Division of AIDS, NIAID, NIH. Strain HIV-1 BaL was originally derived from a primary culture of adherent cells from infant explanted lung. It utilizes CCR5 as co-receptor [22]. HIV-1 MN, which utilizes CXCR4 as co-receptor, was first isolated from blood of a pediatric patient in 1984 [23], and was shown to be positive in cultured cells in the same year [24]. Strain HIV-1 89.6, which utilizes CCR5 and CXCR4 as co-receptors, was originally isolated from a mixed PBMC culture obtained from an AIDS patient and replicates to high titers in primary human macrophages and primary human lymphocytes [25]. The multiplicity of infection (MOI) of HIV-1 BaL, HIV-1 89.6, and HIV-1 MN were determined as described previously [26]. The cells (2×10^6^ cells/ml) were briefly resuspended in medium supplemented with 15% FBS. Virus was added (6 serial dilutions 1∶10), and the plate was incubated at 37°C in humid air containing 5% CO_2_. On the third day, ELISA to detect p24 was done on each well, to establish the Tissue Culture Infective Dose (TCID). After the TCID_50_ was calculated (Spearman-Karber equation), the MOI was obtained and corrected to MOI = 1, for all assays.

### Cytotoxicity Assay

The *in vitro* cytotoxic effect of each of the flavonoids was determined by a resazurin fluorometric method (Cell Titer Blue Viability Assay, Promega Corp, Madison, WI), as described by O'Brien et al. [27]. TZM-bl and HeLa adherent cells were each separately seeded (1×10^5^ cell/mL) into a 96 well plate (Greiner Bio-One North America, Inc Monroe, NC, USA). After 24 hours, their morphology was observed under an inverted microscope. The experimental natural compounds were then added to the cultured cells (n = 9) plates, and incubated for 24 hours. The cells were washed with pre-warmed PBS (Lonza Walkersville, Inc. Walkersville, MD) and the medium was replaced with medium pre-warmed to 37°C. Resazurin was added, and the cells were incubated for 3.5 hours. Fluorescence was read in a microplate reader (SpectraMax M5 Microplate Reader, Molecular Devices, Sunnyvale, CA) with excitation at 550 nm, emission 585 nm, and cut-off 570 nm. The cytotoxic effect of the three flavonoids on H9 and PBMC cells was determined using the same protocol.

### Anti-HIV activity in infected TZM-bl cells

The *in vitro* anti-HIV-1 activity of the flavonoids was determined using a cell-based assay, as described by Ochsenbauer-Jambor et al. [28]. The TZM-bl cells were seeded into a 96 well plate at 1×10^5^ cell/mL. After 24 hours, the flavonoid dilutions or controls, together with HIV-1 BaL (MOI = 1), were added to the cultured cells (n = 9). After 48 hours, the cytotoxic effect was determined by the resazurin fluorometric method. The cells were incubated with resazurin for 3.5 hours, and fluorescence of the supernatant was read in a microplate reader. The attached cells were washed with PBS, and 25 µL/well of the lysis buffer was added to the cell cultures. After 20 minutes at room temperature, the lysed cells were transferred to a new plate, and 20 µL/well of luciferase reagent was added (Luciferase Assay System kit, Promega Corp, Madison, WI). The luminescence obtained was read in a microplate reader (SpectraMax M5 Molecular Devices, Sunnyvale, CA) with 500 ms of integration time.

### Anti-HIV activity in H9 cells exposed to infection with HIV-1 MN or HIV-1 89.6

HIV-1 infection of H9 cells was performed as described by Gurgo et al. [29] as follows: H9 cells were seeded at 1×10^5^ cell/mL into a 24 well plate. After 48 hours, 200 µL/well of new medium together with HIV-1 MN (MOI = 1) or HIV-1 89.6 (MOI = 1), and either the flavonoid dilution to be tested or control vehicle, was added to the wells (n = 9) and the plate was incubated at 37°C, in humid air containing 5% CO_2_. After 48 hours of incubation, the supernatant was collected and frozen at −80°C. ELISA was performed to detect p24, and the cytotoxicity assay was done using the resazurin fluorometric method, as described previously.

### Anti-HIV activity of flavonoids on HIV infection of peripheral blood mononuclear cells (PBMC)

Activated PBMC cells were seeded into a 96 well plate (1×10^5^ cells/mL), as described by Li et al. [21], treated either with flavonoid dilutions or with vehicle controls, infected with HIV-1 MN (MOI = 1) or HIV-1 89.6 (MOI = 1), and incubated at 37°C in humid air containing 5% CO_2_ for 72 hours (n = 9). The viability of the cells was assessed by the resazurin fluometric method as describe previously. After incubation, the supernatant from each well was collected and stored at -80°C for performance of p24 ELISA.

### Quantification of HIV-1 p24 Antigen by ELISA

The HIV-1 p24 Antigen ELISA 2.0 Kit (ZeptoMetrix Corporation, Bufalo, NY) was used to monitor and determine the titer of HIV-1-based lentiviral samples, as described by Gonçalves et al. [30]. For each sample (n = 9), a 225 µL volume was treated with 25 µL of lysing buffer. An ELISA array plate was washed three times with 300 µL/well of wash buffer in a microplate washer (MultiWash III, TriContinent, Grass Valley, CA), and 200 µL/well of each treated samples was transferred to the plate. The plate was incubated at 37°C for 3 hours, then washed six times, incubated for 1 hour with 100 µL/well of HIV-1 p24 detector antibody, washed six more times, and incubated 30 min at room temperature with 100 µL/well of substrate. The reaction was stopped by addition of stop solution (100 µL/well). The absorbance was determined using a microplate reader (SpectraMax M5, Molecular Devices, Sunnyvale, CA) at 450 nm, and the samples were calibrated against the HIV-1 p24 standard curve, generated from a series of standards loaded on the same plate.

### Anti- HIV Reverse Transcriptase Activity

The Reverse Transcriptase Assay is an *in vitro* colorimetric enzyme immunoassay for screening anti-viral agents, and was conducted, as described by Rajote et al. [31] and Tan et al. [32] using the flavonoid Myricetin and controls (n = 6). In a microcentrifuge tube, 20 µL of recombinant HIV-1-RT (Roche Diagnostics, Indianapolis, IN) was diluted in lysis buffer and 20 µL of the appropriate flavonoid dilutions or vehicle control was added and incubated at 37°C. After 1 hour, 60 µL of each of sample was added into a microplate (Roche Diagnostics, Indianapolis, IN) precoated with streptavidin and incubated at 37°C for 1 hour. The wells were washed with washing buffer and 200 µL of anti-DIG-POD (antibody to digoxigenin conjugated to peroxidase) working solution was added to each well and then incubated at 37°C to bind to the digoxigenin-labeled DNA. After 1 hour, the wells were washed and 200 µL of peroxidase substrate ABTS solution was added to each well and was incubated at room temperature for 30 min. The peroxidase enzyme catalyzes the cleavage of the substrate, producing a colored reaction product. The resulting signal intensity is directly proportional to the RT activity and was determined by the absorbance using a microplate reader at 405 nm.

### Microbicide activity of Myricetin in the dual-chamber model

**Figure 2:**
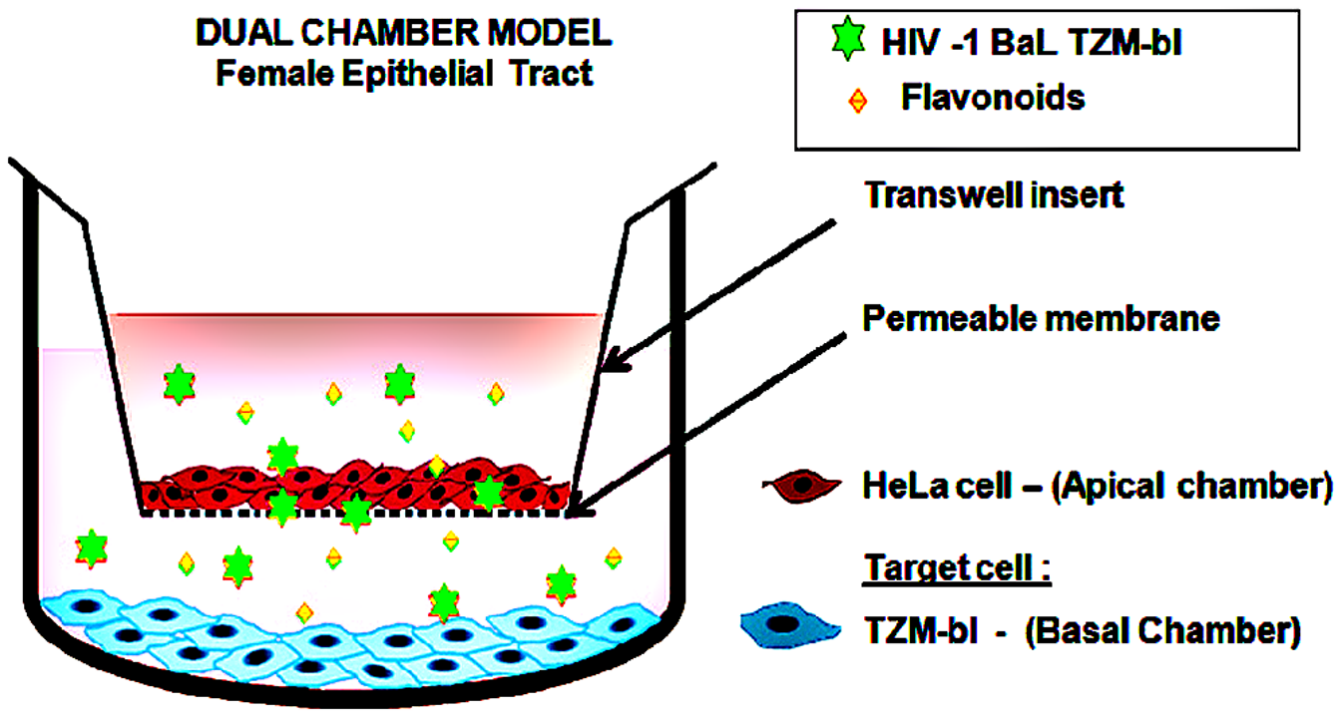
Dual Chamber Model. *In vitro* Dual Chamber Model of female epithelial genital tract for HIV-1 infection and evaluation of candidate microbicides. The apical chamber represents the first barrier of the epithelial layer (HeLa), which is inserted into a 24 well plate (basal chamber) which represents the sub epithelial layer (TZM-bl cell).

The potential microbicide effect of Myricetin was investigated using a dual-chamber system to mimick the epithelium of the female genital tract. (Fig. 2) [33] [34]. Transwell assay inserts with 8 µM diameter pores and 0.3 cm^2^ of culture surface (Greiner Bio-One, Monroe, NC,) were positioned in the wells of a 24-well plate. Then 1×10^4^ HeLa cells were seeded into each transwell insert/apical chamber (using DMEM with 10% FBS). TZM-bl cells (1×10^5^) were seeded into the basal chambers below the inserts, and the plates were incubated at 37°C in humid air containing 5% CO_2_. To assess confluence, the Trans Epithelial Electric Resistance (TEER) (described by Arien et al. [33]) of each HeLa cell layer was measured with a Millicell-ERS Volt-Ohm Meter (Millipore, Bedford, MA) before and after flavonoids treatments. Cell layer confluence in the transwell inserts was measured daily, until the optimal TEER (>150 Ohm/cm^2^) was reached on day 4. On day 4, the media in the basal chamber and the insert was replaced with new pre-warmed media. In the apical chamber/transwell inserts, the flavonoid, Myricetin, or vehicle control was added with the HIV-1 BaL (n = 9). The plate was incubated at 37°C, in humid air containing 5% CO_2_. After 24 hours (day 5), the cell viability and Luciferase assays were performed to analyze the cytoxicity and anti-HIV activity of Myricetin in TZM-bl cells.

### Statistical Analysis

The experiments were performed in triplicate and repeated three different times. The statistical analysis was performed using raw data. To analyze viability, data of the experimental groups were compared to the negative control (only cells). To analyze infection inhibition the data were compared to the vehicle control. One-way Analysis of Variance followed by the Dunnett test or the Dunn test was performed (alpha = 0.01). using JMP software (version Pro 11.0.0; SAS Institute Inc.). Non Linear regression was done, to determine the LD_50_ and the IC_50_, using five parameter logistics with the MasterPlex 2010 Reader Fit (Hitachi Solutions America, Ltd, San Bruno, CA.).

## Results

### Cytotoxicity

**Figure 3:**
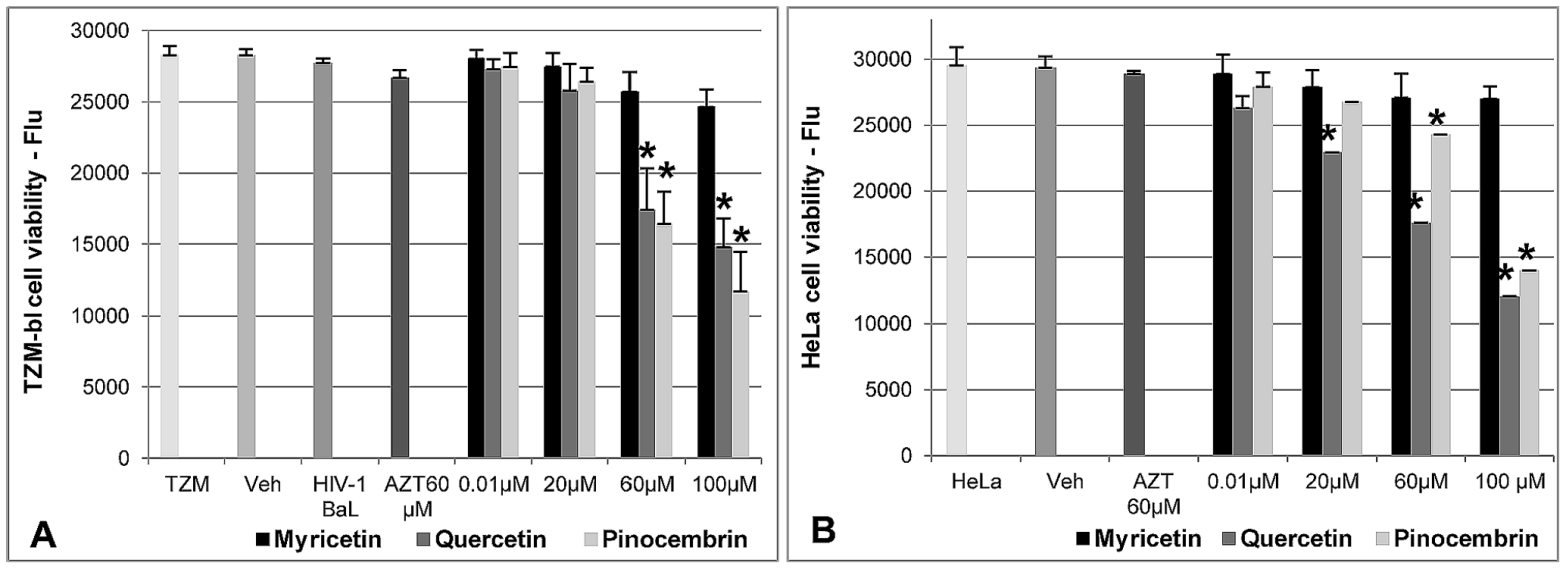
Natural compound toxicity. Viability of **A**) TZM-bl cells and **B**) HeLa cell after 24 hours of treatment with different concentrations of Myricetin (range: 0.01–100 µM), Quercetin and Pinocembrin. (n = 9). TZM: negative control (only cells); Veh: vehicle control and AZT: positive control. Values shown with an asterisk (*) are statistically significant, when compared with the negative control (only cells).

**Figure 4:**
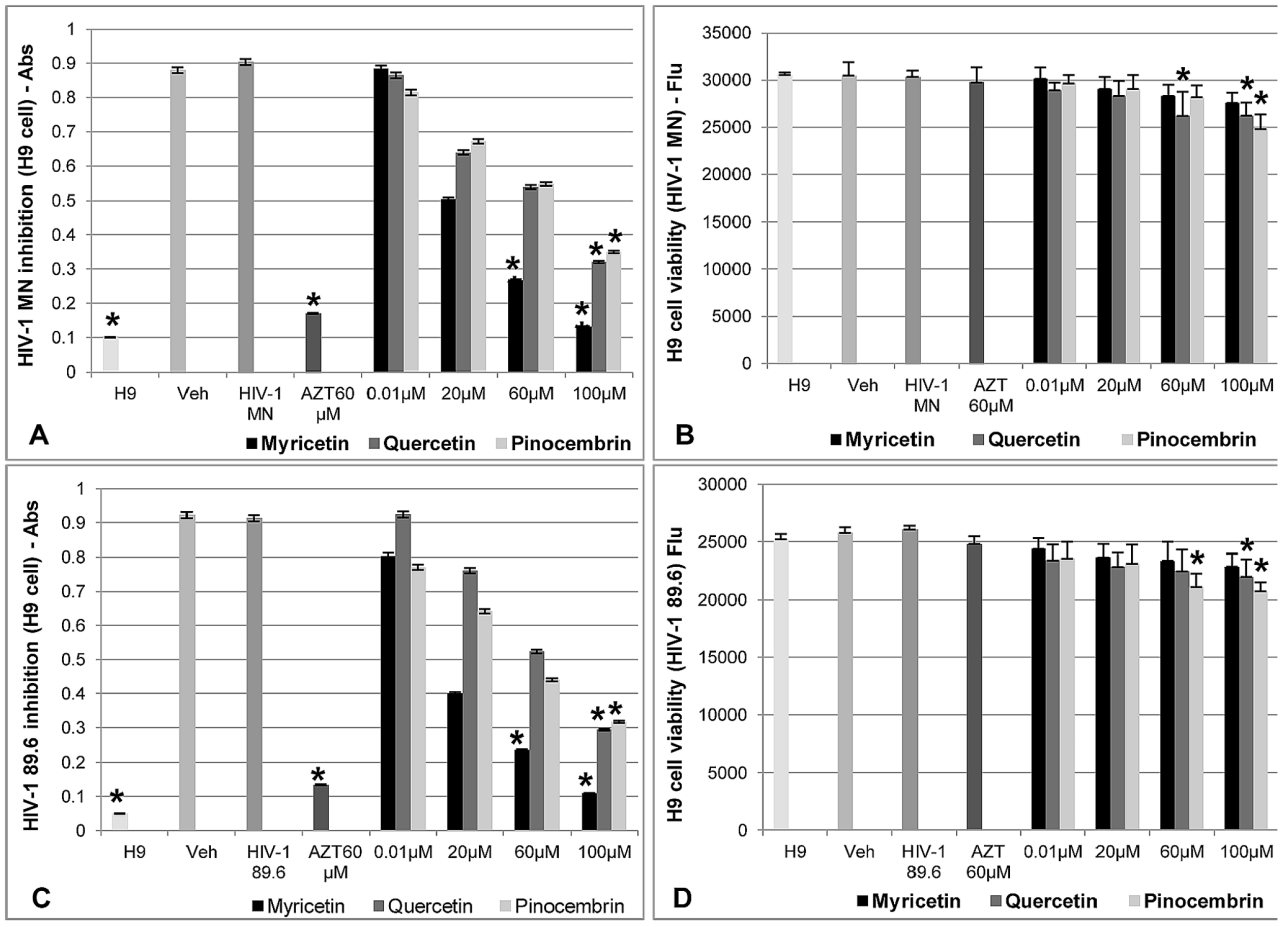
Effects of Myricetin, Quercetin, and Pinocembrin on HIV-1 MN and HIV-1 89.6 infection in H-9 cells and on cell viability. **A, C**) quantification of HIV-1 p24 protein assessed by ELISA, **B, D**) cell viability after flavonoids treatment (n = 9). AZT: positive control; H9: negative control (only cells); HIV-1 MN and HIV-1 89.6: negative control (virus) and Veh: vehicle control. Values shown with an asterisk (*) are statistically significant, when compared with the vehicle control (infection inhibition) and H9 cells viability (negative control: only cells).

**Figure 5:**
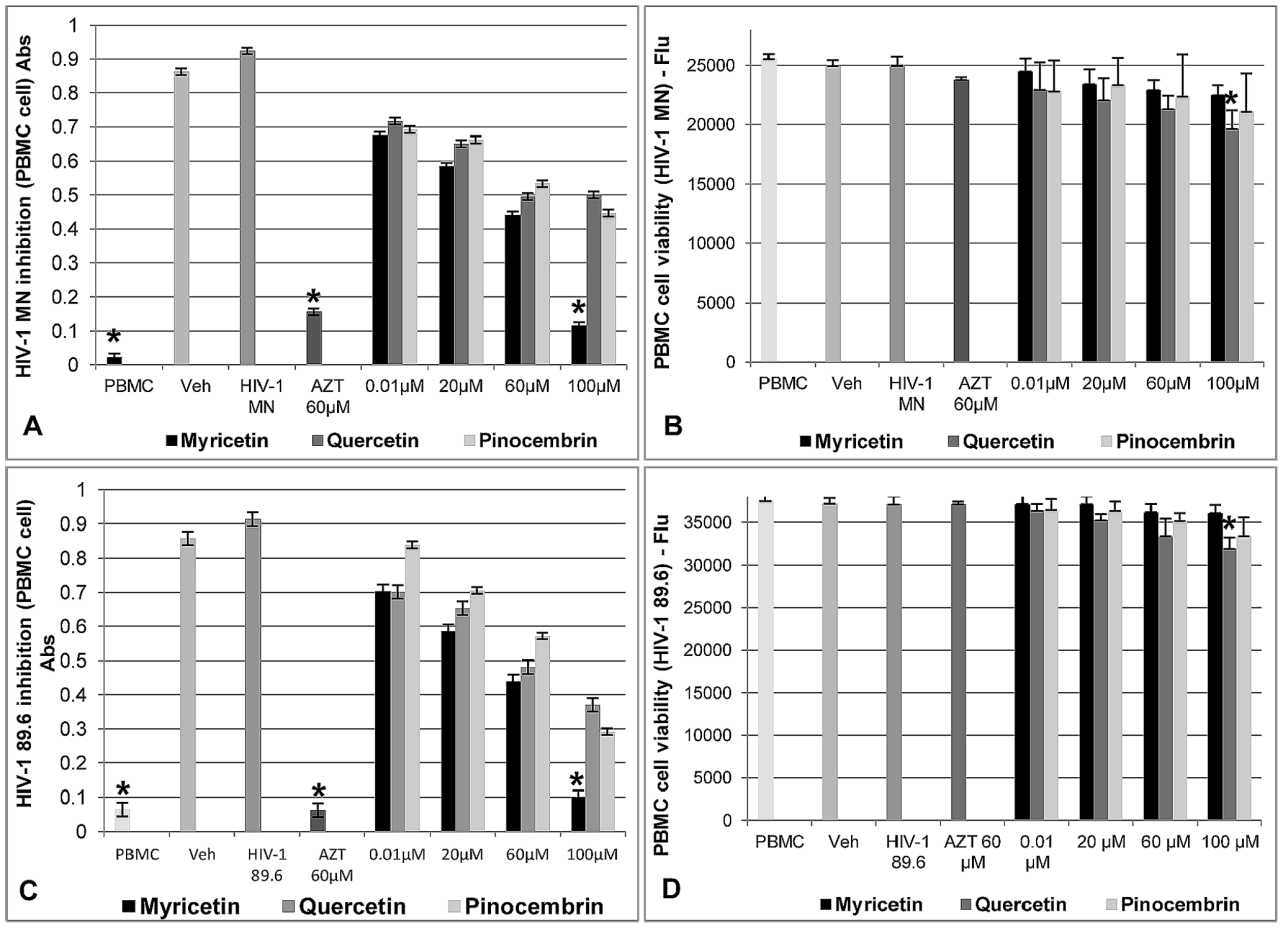
Effects of Myricetin, Quercetin, and Pinocembrin on HIV-1 MN and HIV-1 89.6 infection in PBMC cells and on cell viability. **A, C**) quantification of HIV-1 p24 protein assessed by ELISA **B, D**) cell viability after flavonoid treatment (n = 9). AZT: positive control; PBMC: negative control (only cells); HIV-1 MN and HIV-1 89.6: negative control (virus) and Veh: vehicle control. Values shown with an asterisk (*) are statistically significant, when compared with the vehicle control (infection inhibition) and PBMC cells (negative control) (cell viability).

Cytotoxicity tests were conducted on TZM-bl, HeLa, PBMC, and H9 cells. The LD_50_ of the flavonoids were summarized on Table 1. The Myricetin presented ≥85% of cell viability at 100 µM concentration when compared with the negative control (only cells). Quercetin and Pinocembrin presented ≥85% cell viability at concentrations between 20 µM–60 µM. The positive (AZT) and vehicle controls (1% DMSO) did not affect the cell growth. The results are shown in Figs. 3 (A, B), 4 (B,D) and 5 (B,D).

**Table 1:** Cell viability after flavonoids exposition.

LD_50_ (µM)			
Cell Line	Myricetin	Quercetin	Pinocembrin
TZM-bl	1214.72	119.23	67.94
HeLa	804.94	78.20	99.52
PBMC	1159.26	813.57	788.23
H9	1059.09	905.76	805.76

The LD_50_ values (means the concentration that kill 50% of the cells) were determinate from nonlinear regression analysis by MasterPlex 2010 Reader Fit.

### Anti HIV activity

**Figure 6:**
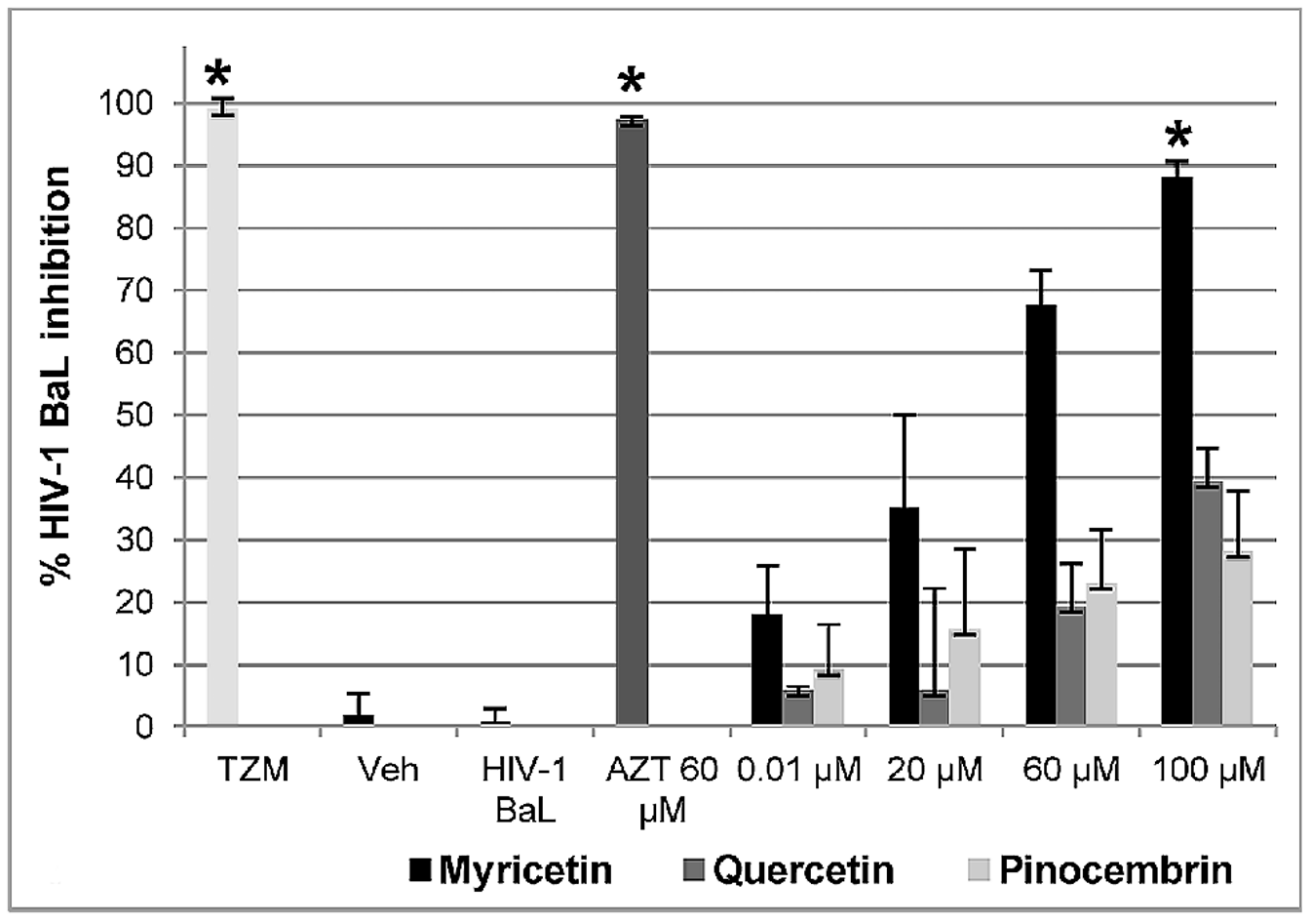
Anti HIV-1 activity of the flavonoids on TZM-bl cells. Anti-HIV activity was monitored by luminescence of luciferase (TZM-bl reporter cells) after 24 hours of treatment with Myricetin, Quercetin and Pinocembrin (range: 0.01–100 µM) (n = 9). AZT: positive control; TZM: negative control (only cells); HIV-1 BaL: negative control (virus) and Veh: vehicle control. Values shown with an asterisk (*) are statistically significant, when compared with the vehicle control.

Respectively, Quercetin (IC_50_ 88.98 µM) and Pinocembrin (IC_50_ 346.75 µM) showed 39% and 28% anti-HIV activity, when compared with the vehicle control. At 100 µM, Myricetin inhibited >87% of HIV-1 BaL infection in TZM-bl cells (IC_50_ 20.43 µM). The positive control (AZT) inhibited ≥90% the HIV infection, and the vehicle control (1% DMSO) did not affect the infectivity of the HIV-1(Fig. 6).

The IC_50_ of all experiments that analyzed the anti-HIV activity of the flavonoids are summarized on Table 2.

**Table 2:** Inhibitory activity of HIV-1 BaL, HIV-1 MN and HIV-1 89.6 by flavonoids.

IC_50_ (µM)				
Cell line	Strain	Myricetin	Quercetin	Pinocembrin
TZM-bl	BaL	20.43	88.98	346.75
H9	MN	22.91	38.78	67.20
H9	89.6	1.76	29.76	42.20
PBMC	MN	4.49	31.68	57.67
PBMC	89.6	3.23	39.26	53.20

The IC_50_ values (means the concentration that inhibit the 50% of HIV-1 infection) were determinate from nonlinear regression analysis by MasterPlex 2010 Reader Fit.

The results of p24 quantification by ELISA, after H9 cells were treated with the flavonoids and exposed to HIV-1 MN or HIV-1 89.6, showed that Myricetin presented IC_50_ 22.91 µM and 1.76 µM, respectively, and inhibited the infection by ≥86%. Quercetin presented ≥64% (IC_50_ 38.78 µM; 29.76 µM) and Pinocembrin presented ≥60% (IC_50_ 67.20 µM; 42.20 µM) of anti-HIV activity (Fig. 4–A, C).

When anti-HIV activity in PBMC cells was analyzed by p24 ELISA, it indicated that Myricetin (100 µM) inhibited 86% of the HIV-1 MN infection, (IC_50_ 4.49 µM), and 85% (IC_50_ 3.23) of the HIV-1 89.6 infection. Quercetin and Pinocembrin presented, respectively, 45% and 59% (IC_50_. 31.68 µM; 39.26 µM) and 67% and 51% (IC_50_. 57.67 µM; 53.2 µM) anti-HIV activity with both strains of virus (Fig. 5–A, C).

Since Myricetin presented the best results in all of the preceding experiments, we decided to investigate both the anti-HIV-RT and microbicide effects of this flavonoid. The anti-HIV-RT was investigated using a reverse transcriptase assay and the microbicide effects was studied using a dual chamber model of the female genital tract.

### Anti-HIV RT (Reverse Transcriptase) Activity of Flavonoids

**Figure 7:**
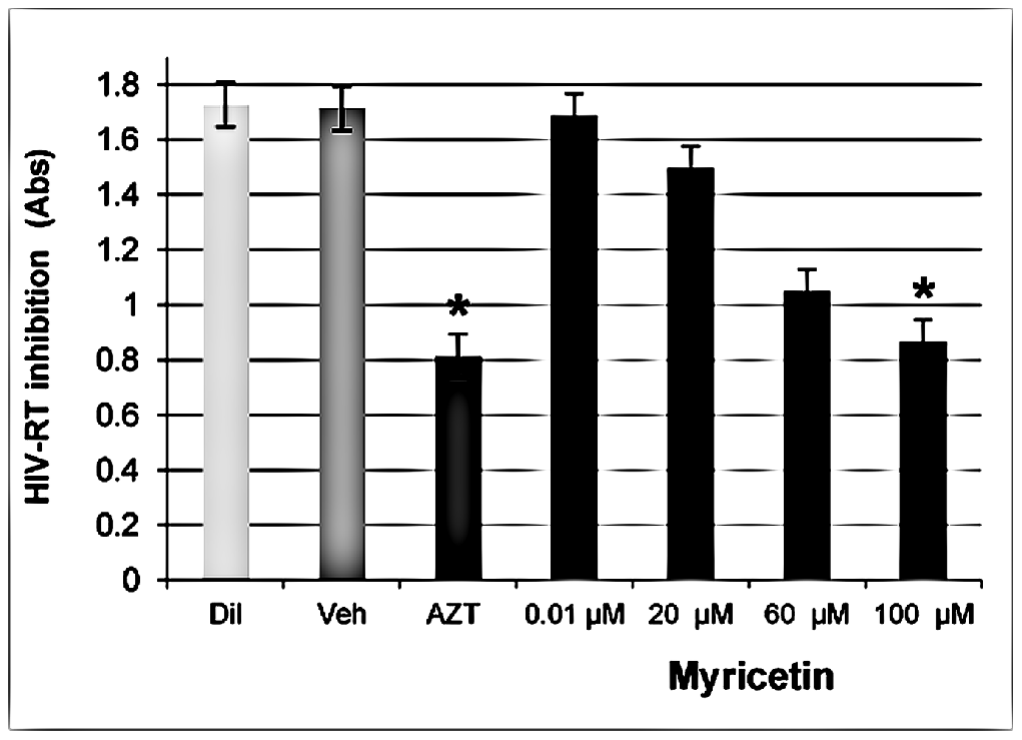
Anti HIV-1–RT effect of Myricetin IC_50_ 203.65 (n = 6). The positive control used was 60 µM AZT, and the negative controls were Vehicle (1%) and diluent substance without Myricetin and vehicle. Values shown with an asterisk (*) are statistically significant, when compared with the negative control (without flavonoid and vehicle).

Quantification of the inhibitory effect on HIV reverse transcriptase was done for Myricetin (0.01–100 µM) only, and the results are shown in Fig. 7. The inhibitory activity of Myricetin (IC_50_ 203.65 µM) against HIV-1 reverse transcriptase was the greatest, ≥49%, at the 100 µM concentration. The results were compared with the negative control.

### Microbicide activity of Myricetin using the Dual Chamber System

**Figure 8:**
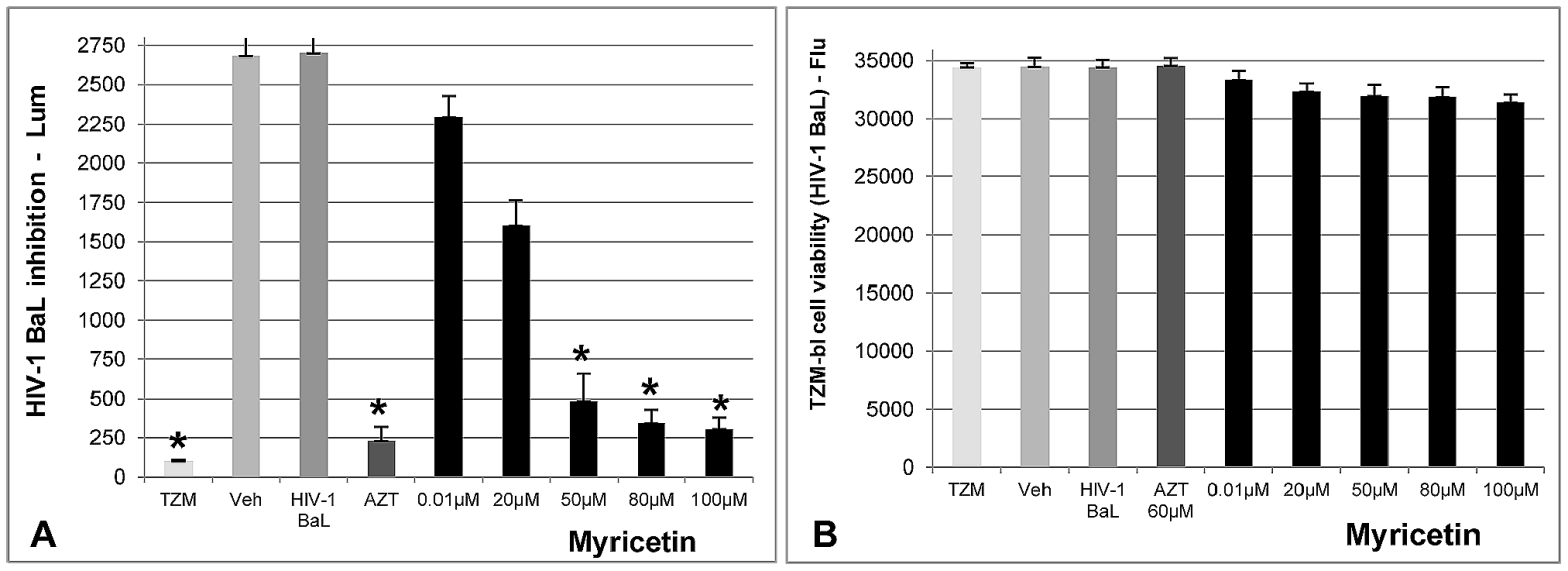
Myricetin-mediated inhibition of HIV-1 BaL infection in TZM-bl cells and effect on cell viability in Dual Chamber Model. The microbicide activity of Myricetin and cell viability was assessed by Luciferase assay and resazurin method, respectively. **A**) Microbicide activity of Myricetin (IC_50_ 19.51); **B**) Cell viability after Myricetin treatment (n = 9). Myricetin treatment did not show a statistically significant difference on cell viability. AZT: positive control; TZM: negative control (only cells); HIV-1 BaL: negative control (virus) and Veh: vehicle control. Values shown with an asterisk (*) are statistically significant, when compared with the vehicle control (microbicide activity) and TZM-bl cells (cell viability).

The HeLa cells cultured on the transwell insert formed a confluent layer, demonstrated a TEER measurement of 208 Ohm/cm^2^ both before and after flavonoid treatment. Therefore the Myricetin treatment did not affect the confluence layer. In the absence of inhibitors, the HIV-1BaL virus crossed this HeLa cell layer to the basal compartment, infecting the target TZM-bl cells. Myricetin treatment inhibited the HIV-1 BaL infection in this system, showing an IC_50_ of 19.51 µM, and more than 88% inhibition at 100 µM concentration. These results were obtained by the Luciferase assay method on day 6, following the introduction of Myricetin and the HIV-1 BaL. The cell viability (resazurin method) was also determined on day 6, and a there was no statistically significant difference between the negative control (only cells) and the treatment (Myricetin) as shown in Fig. 8–A, B.

## Discussion

Natural products continue to be major sources of innovative therapeutic agents for treatment of infectious diseases, and their exploration has been one of the most successful strategies for the discovery of medicines [35]. Flavonoids are phytochemical compounds, commonly found in plants, and some of which have been used for centuries as active compounds in medicinal preparations to treat human diseases [36]. Structurally, the flavonoids are polyphenols with diphenylpropane (C6-C3-C6) skeletons [16], resembling folic acid and nucleosides [15], [16]. The structure of Myricetin presents 3′, 4′, and 5′ hydroxyl groups, whereas Quercetin presents only two adjacent hydroxyl groups at the 3′ and 4′ positions, and Pinocembrin does not present hydroxyl groups at any of these positions (Fig. 1A, B, and C).

Several flavonoids show anti-viral [13], [37], [38], anti-oxidant [39]–[41] anti-inflammatory [42], [43], anti-bacterial [37], [38], [43], [44] and anti-cancer [45], [46] activities. The microbicide effect mechanism by which flavonoids function to inhibit HIV infection is unknown. However, our reported data can provide some insights into their inhibitory action.

The cytotoxicity of Myricetin, Quercetin, and Pinocembrin evaluated in TZM-bl, HeLa, PBMC, and H9 cells show overall data indicating that these flavonoids had low toxicity. Myricetin had the lowest toxic effect on the tested cells, with LD_50_ 1214.92 µM in TZM-bl, LD_50_ 804.94 µM in HeLa, LD_50_ 1158.66 µM in PBMC, and LD_50_ 1059.09 µM in H9 cells. Our data support the findings reported by Mehla et al. [17] showing that flavonoids Myricetin and Quercetin have low toxicity in Jurkat and TZM-bl cells.

To determine the anti-HIV activity of flavonoids, this study used dual tropic, T-tropic and M-tropic strains of HIV-1. In general, the three flavonoids were active against HIV-1 BaL (R5 tropic virus), whereas Myricetin presented the highest anti-HIV-1 activity (IC_50_ 20.43 µM), with more than 87% of infection inhibited at 100 µM. The IC_50_ of Myricetin for HIV1 BaL showed that this flavonoid inhibited HIV-1 infection, approximately 4-fold more effectively than Quercetin, and 16-fold more effectively than Pinocembrin. These results are in accordance with [17] which showed that the flavonoids, Myricetin and Quercetin, had anti-HIV activity on TZM-bl cells. The anti-HIV activity of flavonoids against M-tropic and -tropic virus was tested, using H9 cells infected by HIV-1 MN (T-tropic) and HIV-1 89.6 (dual tropic). Myricetin was the most effective inhibitor of HIV-1 infection in this context, presenting 85% inhibition of HIV-1 MN, with IC_50_ 22.91 µM, and 88% inhibition of HIV-1 89.6, with IC_50_ 1.76 µM. The anti-HIV activity of Myricetin against HIV-1 MN was approximately 1.7x greater than that of Quercetin, and 2.9x greater compared to Pinocembrin. The anti-HIV activity of the Myricetin in H-9 cells exposed to HIV-1 89.6 was 16.9x greater than for Quercetin, and 23.9x greater than for Pinocembrin. Moreover, we evaluated the anti-HIV activity of these flavonoids on human peripheral blood mononucleated cells (PBMC) that express CCR5 and CXCR4 co-receptors. Myricetin inhibited HIV-1 MN with IC_50_ 4.49 µM, which was 7-fold more effective than Quercetin, and 12-fold more effective than Pinocembrin. In PBMC cells infected by HIV-1 89.6, Myricetin presented an IC_50_ of 22.91 µM, 12.3x higher than Quercetin, and 16.4x higher than Pinocembrin. It is noteworthy that Myricetin possess anti-HIV activity comparable to the positive control (AZT) on dual-tropic, T-tropic, and especially on an M-tropic strain, which is the most prevalent type of HIV-1 strain in under-developed countries such as South Africa and India [47].

To elucidate the mechanism of the Anti-HIV effects of Myricetin, the compound's inhibition of HIV-1 reverse transcriptase enzymatic activity was tested. The HIV-1 RT is unique to the virus, and is the enzyme that controls HIV-1 replication infected cells [4], [48]. We found that Myricetin inhibited HIV-1-RT activity. Our results are in accordance with recent studies [13], [48], showing that Myricetin possess inhibitory activity against HIV-1 viral enzymes.

It is important to note that flavonoids, beyond action against reverse transcriptase, also modulate several steps of HIV-1 life cycle, including entry, integration and maturation phases [49], [50]. Flavonoids can inhibit the HIV entry by affecting the receptor CD4 and co-receptors CXCR4 and CCR5 [51], therefore blocking the communication from a cell's surface sensors to its interior (Xu et al., 2013), and the integrase enzymes [52], [53]. Flavonoids can reverse the expression profile of some cellular genes involved in protein kinase C, survival, stress and apoptosis signaling pathways that are altered during the viral infection. These modulations include the down regulation of IL-2 and IL-2R alpha in the kinase C pathways [54], [55], the up regulation of the inhibitor of apoptosis proteins IAP2 (BIRC2) and IAP1 (BIRC3) in the survival pathway [56], the up regulation of the heat shock proteins hsp27 (HSPB1), hsp90 (HSPCA) in the stress pathway and the up regulation of the BCL2-associated X protein Bax, and the down regulation the DNA-damage-inducible transcript gadd45, in apoptosis pathway [56]. Flavonoids can modulate the protein transcriptional factors NF-Κβ and NFAT too [17], [55].

Overall, the flavonol, Myricetin, with adjacent hydroxyl groups at the 3′, 4′, and 5′ positions, showed superior inhibition of HIV activity compared to the other two flavonoids that presented only modest activity against HIV-1: Quercetin, which lacks a hydroxyl group at the 5′ position, and Pinocembrin that lacks hydroxyl groups at the 3′, 4′, and 5′ positions. Results from previous studies by Mehla et al. [57] have shown that the flavonoid, Luteolin, which lacks hydroxyl groups on 5′ and 3′ positions, presents higher toxicity and activity against HIV-1 than Myricetin, Quercetin or Pinocembrin. These observations suggest that a hydroxyl group at position 3 is required for inhibitory effects, and additionally, the hydroxyl groups at 3′, 4′, and 5′ affect the toxicity.

The potential microbicide activity of Myricetin was evaluated by the dual-chamber model [33], [34], [58]. Myricetin has shown two important requirements for a product to become a microbicide, as according to WHO regulatory guidelines for microbicide development [10]. The first requirement is low toxicity. Myricetin presented high therapeutic index (137.46), and low cytotoxicity on eukaryotic cell, indicating that it is safe. The second requirement is the inhibitory activity on HIV infection. Myricetin demonstrated anti-HIV-1 activity in the dual-chamber model, and confirmed our previous results for anti-HIV tests with different strains of HIV-1 (BaL, MN and 89.6). Therefore, Myricetin presented, in our *in vitro* study, two critical characteristics to be considered as a potential microbicide.

The development of new therapeutic agents that act as microbicides are considered the most promising preventive interventions in AIDS research [8], [59]. Several microbicides are in various stages of testing, such as Caprisa 004, which reduces the risk of contracting HIV during sex by 39% [8], [60]. The prevention of infection, by sexually transmitted HIV-1, is an important way to reduce the number of cases of HIV infection around the world [6], [59].

Therefore, this study, having determined the anti-HIV activity of flavonoids against different strains of HIV-1, reports for the first time the promising *in vitro* potential microbicide activity of Myricetin. Nevertheless, further studies with Myricetin are still needed in order to identify the molecular targets and biological signatures of this and other flavonoids. Such studies will provide the solid biological foundation for translational research, which is needed to evaluate the *in vivo* activity of Myricetin and related compounds.
